# Impacts of different biochar types on the anaerobic digestion of sewage sludge

**DOI:** 10.1039/c9ra08700a

**Published:** 2019-12-20

**Authors:** Min Zhang, Jianhua Li, Yuncai Wang, Changming Yang

**Affiliations:** Department of Landscape Architecture, Center for Ecophronetic Practice Research, College of Architecture and Urban Planning, Tongji University 1239 Siping Road Shanghai 200092 China wyctongji@163.com +86-21-65986707 +86-21-65980253; Key Laboratory of Yangtze River Water Environment of the Ministry of Education, Tongji University 1239 Siping Road Shanghai 200092 China cmyang@tongji.edu.cn +86-21-65986313 +86-21-55962975

## Abstract

In this study, the effect of nine types of biochar generated from three different feedstocks on the anaerobic digestion (AD) of sewage sludge was investigated. The obtained results indicated that methane production could be significantly enhanced by all types of biochar used in the test. The maximum cumulative methane yield of 218.45 L per kg VS was obtained for the culture with corn straws pyrolyzed at 600 °C which also exhibited the largest specific surface area. Adding an appropriate amount of biochar was beneficial to improve the cumulative methane yield, while excessive addition could inhibit the AD process. Biochar could also enhance AD process stability by increasing buffering capacity, releasing volatile fatty acid accumulation and alleviating ammonia inhibition. Simultaneously, microbial community analysis revealed that biochar addition was able to improve the diversity of archaeal community and adjust the microbial communities. It was notable that biochar treatment facilitated the aceticlastic methanogens (*Methanosarcina*) compared to the hydrogenotrophic methanogens. Overall, biochar addition could be an ideal approach that is not only expected to successfully improve the performance of AD, but also lay a new path for future biomass energy utilization.

## Abstract 2

Pyrolysis temperature and feedstock types had a pronounced effect on biochar properties, and biochar could facilitate the anaerobic digestion process.

## Graphical abstract

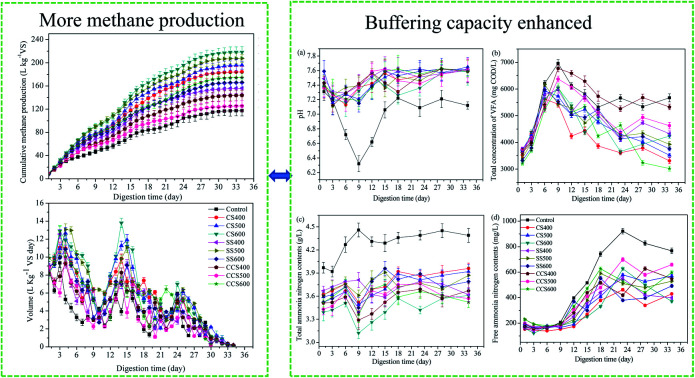

## 1. Introduction

A large quantity of sewage sludge is produced from municipal wastewater treatment plants (WWTPs) annually due to the expansion of population and industry.^1^ So, sludge treatment and disposal problems need to be solved for environmental protection. Sewage sludge is rich in organic matter and other easily available nutrients for plant tissues, which has great potential as a fertilizer for agriculture due to the many benefits it provides, including enhancing soil fertility and improving nutrient status.^2,3^ Meanwhile, sewage sludge also contain heavy metals and other pollutants such as pathogens, persistent organic compounds and so on.^4^ Accumulation of high concentrations of toxic heavy metals in sewage sludge might pose a threat to public health by their entering into the ecological cycle, due to their difficult biodegradability and harmful nature.^5^ Hence, WWTPs now need to employ appropriate treatment technology to meet the tighter governmental regulations prior to discharge of sludge to the land.

Anaerobic digestion (AD), a cost-effective and widely used method for treating sludge, is a complicated microbial process where organic wastes are transformed into stable macromolecules by hydrolysis, acidogenesis, acetogenesis and methanogenesis, which occur sequentially.^6^ AD has been recommended as one of the most promising technologies for stabilization of sewage sludge, leading to sludge reduction, biogas production and green energy recovery. However, AD of sewage sludge is widely limited by the intermediates and by-products produced in the AD process, causing low methane yield, process instability and various inhibition problems.^7,8^ Based on the above considerations, some works proposed several technologies based on AD process and performance efficiency, such as thermal hydrolytic pretreatment,^9^ microwave-H_2_O_2_ pretreatment,^10^ anaerobic co-digestion with different substrates,^11^ and the addition of metal nanoparticles like cobalt (Co), iron (Fe) and nickel (Ni), with the aim of improving digester efficiency.^12–14^ Considerable studies in recent years have revealed increasing interest in carbon-based conductive materials such as granular activated carbon (GAC),^15^ carbon nanotubes^16^ and biochar, because their additions are very helpful in stimulating the AD process. Additions of carbon-based materials are effective to improve digestate quality because they could facilitate the direct interspecies electron transfer process, promote microbial immobilization and metabolism,^17^ increase fertilizer nutrient retention and alleviate the accumulation and inhibition of interspecific products.^18^ Dang^19^ illustrated that methane yield and chemical oxygen demand (COD) removal efficiencies in a GAC-supplemented reactor were almost 17.85 and 1.5 times more than those of controls. Mostafa^20^ found that the degradation efficiency of organic compounds (carbohydrates, lipids and proteins) and hydrogenase enzyme activity were all significantly higher in magnetite/graphene oxide-amended reactors than that in the control. Besides, Jang^21^ studied the effects of dairy manure-derived biochar on the AD of sludge and found 24.9% more methane production with 10 g L^−1^ biochar supplement.

Biochar is a novel carbonaceous porous material produced by thermochemical conversion or pyrolysis of biomass with little or no oxygen.^22^ Recently biochar has attracted increasing attention in several engineering applications due to its distinctive characteristics such as large surface area,^23^ porous structure, oxygenated functional groups and cation exchange capacity.^24^ Biochar as an additive in AD systems could improve microorganism metabolism and optimize the structure of microbial communities, alleviate inhibitor stress and maintain AD process stability.^25,26^ Ma^27^ investigated the effects of biochar addition on the semi-continuous AD of chicken manure at mesophilic temperature. The results showed that biochar supplementation not only accelerated the degradation of propionic acid, but also enhanced AD buffering capacity, further resulting in higher methane yield. Shen^28^ noticed that cultures with biochar supplementation simultaneously enhanced the methane content in the biogas and macro-nutrients in the digestate in comparison to the control. According to Alves,^29^ biochar addition could effectively increase long-chain fatty acid decomposition and shorten the lag phase during the AD process. Duan^30^ found that supplement of algae-derived biochar to algae anaerobic fermentation raised the hydrolysis efficiencies of organic compounds (polysaccharide, proteins and lipids) by 120–140% compared to that without biochar. Additionally, studies evidenced that the raw materials and pyrolysis temperature employed in carbonization can directly influence biochar properties such as the sorption capacity, ion-exchange capacity and catalytic activity.^31^ For example, Fagbohungbe^32^ evaluated three different types of biochar (wood biochar, coconut shell biochar and rice husk biochar) in the AD of citrus peel waste and the results suggested that coconut shell biochar achieved the highest methane production while the addition of wood biochar induced the shortest lag phase. As far as we know, although the promotion of the AD process by biochar supplementation has been widely reported, studies on the impacts of biochar derived from different feedstocks under various pyrolytic temperatures on AD process stability are limited.

In this regard, this work aims to evaluate the effectiveness of biochar derived from three different feedstocks under three different pyrolytic temperatures in the AD of sewage sludge under batch mode at the laboratory scale. The first aim was to evaluate the effect of different types of biochar and biochar dose addition on methane production; the second aim was to study the effect of biochar addition on the enhancement of AD system stability. Finally, the effectiveness of different types of biochar on the microbial community with high-throughput sequence technology was also evaluated. The findings of this study provide valuable information regarding biochar addition to improve the performance of the AD process.

## 2. Materials and methods

### 2.1. Biochar production

Three different types of biomass were used as raw materials for biochar preparation in the study. Corn straw (CS) was collected from a local cropland in Shanghai, coconut shell (CCS) was purchased from a local farmers' market in Shanghai, and sewage sludge (SS) was obtained from a local municipal WWTP in Hefei City of Anhui Province. Pyrolysis temperature is an important factor that affects the physicochemical properties of biochar and research shows that pyrolysis performed at temperatures of 350–600 °C is beneficial for biochar stability.^31^ The feedstocks were air-dried at room temperature, smashed and then pyrolyzed using a tube furnace in oxygen-free conditions. Then they were pyrolyzed at 400 °C, 500 °C and 600 °C for 120 min, with a heating rate of 15 K min^−1^. After the pyrolysis, the biochar samples were ground and sieved to a 100-mesh sieve. The prepared biochar was then dried at 105 °C and vacuum stored at 4 °C for subsequent use. Nine biochar samples were abbreviated as CS400, CS500, CS600, CCS400, CCS500, CCS600, SS400, SS500 and SS600, respectively, according to the highest pyrolysis temperature.

### 2.2. Biochar characterization

The pH of the different types of biochar was measured in a 5% (w/v) biochar/distillate water suspension after 24 h of stirring at 160 rpm. pH was determined using a pH meter (PHS-3C, Lei-Ci, Shanghai, China). The determination of carbon, nitrogen, oxygen and hydrogen content of the samples was conducted using an isotope ratio mass spectrometer (IRMS, IsoPrime 100, Elementar, Germany). The specific surface area of biochar samples was analyzed *via* an ASAP2020 instrument (Micromeritics, Norcross, USA) using N_2_ sorption isotherms and the Brunauer–Emmett–Teller (BET) method.^33^ The ash content of biochar samples was determined by combustion of dried samples to constant weight in a muffle furnace at 650 °C for 120 min, and then calculating the mass residual percentage of the samples.

### 2.3. Substrate and inoculum sources

The sludge samples for mesophilic AD experiments were obtained from a municipal WWTP in Hefei City of Anhui Province. The digested (inoculum) sludge samples were obtained from an anaerobic digester that has been steadily operated under mesophilic conditions in the National Engineering Research Center for Urban Pollution Control of Tongji University. The main characteristics of raw sewage sludge and inoculum are presented in Table 1. The substrates were stored in a refrigerator at 4 °C for subsequent use.

**Table 1:** Main characteristics of raw sludge and inoculum

Parameter	Substrate	Seed sludge
TS (%)	11.6 ± 0.2^a^	9.7 ± 0.3
VS (%)	56.9 ± 1.7	49.2 ± 1.1
pH	6.7 ± 0.1	6.8 ± 0.2
SCOD (mg L^−1^)	692.9 ± 17.9	7216.9 ± 216.7
TCOD (mg L^−1^)	39 092.7 ± 921.6	86 276 ± 1239.2
Soluble proteins (mg L^−1^)	10.9 ± 0.3	239.6 ± 5.6
Soluble carbohydrates (mg L^−1^)	79.2 ± 1.7	339.6 ± 9.2

a Values are expressed as the mean ± standard deviation (SD).

### 2.4. Anaerobic digestion

500 mL glass bottles were employed as reactors for batch AD experiments. Raw sludge and inoculum were well homogenized at a ratio of 3 : 1 (based on the VS) and then diluted to 10% of the total solid. In order to evaluate the effect of different types of biochar on AD, 8 g L^−1^ of each biochar was added to the reactors. The biochar dose was selected according to the results obtained from a previous study^34^ and orthogonal experiments conducted as described previously. Preliminary results indicated that the biochar dose was optimum for methane production and stability of the AD of sewage sludge. Together, there were 9 groups of biochar-amended bottles (CS400, CS500, CS600, CCS400, CCS500, CCS600, SS400, SS500 and SS600) and one control group without biochar addition. Before experiments, all digesters were flushed with pure N_2_ (>99.99%) for 3 min and subsequently filled with 300 mL of sludge mixtures and biochar and then sealed with rubber plugs.^35^ All reactors were then placed in thermostatic shakers maintained at 70 rpm agitation (35 °C) until gas production stopped, within approximately 34 days. In addition to biochar types, the effects of biochar dose on AD performance were also studied. Five different biochar addition ratios of blank, 6.2 g L^−1^, 15.9 g L^−1^, 26.1 g L^−1^ and 34.2 g L^−1^ were set up, for which the daily and cumulative methane production were measured. All the AD experiments were carried out in batch mode. Each set of the experiments in the study were conducted three times and the average values were reported.

### 2.5. Chemical analyses

Total solids (TS), volatile solids (VS), TCOD, soluble chemical oxygen demand (SCOD), alkalinity and ammonia nitrogen were determined by using standard methods.^36^ The free ammonia nitrogen (FAN) was calculated as reported by Hansen.^37^ The pH was determined by a pH meter (model: PHS-3C, Leici Co. Ltd, Shanghai, China). Biogas composition was determined by gas chromatography (Agilent Technologies 6890N, CA, USA) facilitated with a thermal conductivity detector. The daily biogas volume produced from each reactor was measured using a gas sampling bag (Asone, Japan). The total and individual concentrations of volatile fatty acids (VFAs) were determined using a gas chromatograph (Shimadzu GC, 2014) equipped with a hydrogen flame detector. Sludge samples were collected from each digester on day 0, 3, 6, 9, 12, 15, 18, 23, 28 and 33 to analyze physicochemical parameters.

### 2.6. DNA extraction, PCR amplification and sequencing analysis

High-throughput sequencing was carried out on an Illumina platform (Illumina Miseq PE250) of Novogene Technology Co. Ltd of China. DNA was extracted from the sludge samples using a PowerSoil DNA extraction kit (MoBio, USA) in accordance with the standard protocols. The primer sets 787F (5′-ATTAGATACCCSBGTAGTCC-3′) and 806R (5′-GGACTACCAGGGTATCTAAT-3′) were used to amplify the V4 regions of the bacterial 16S rRNA gene. And the primer sets 518F (5′-CCAGCAGCCGCGGTAATACG-3′) and 805R (5′-GACTACCAGGGTATCTAATCC-3′) were used for the archaeal 16S rRNA gene. After purification and quantitation, pair-end sequencing was performed with an Illumina MiSeq PE250 system. All the qualified sequences were binned into operational taxonomic units (OTUs) based upon 97% similarity. Final taxonomical assignment was conducted based on the MIDA database version 2.1.

### 2.7. Statistical analysis

In this work, one-way analysis of variance was used to test the significant differences of the experimental results. Duncan's multiple range test was used to compare the differences between the means of the treatments. Significant differences between values were assumed at *p* < 0.05. All statistical analyses were performed in SPSS 16.0 and Origin 9.0 for windows.

## 3. Results and discussion

### 3.1. Effect of feedstock types on characteristics of biochar

The physical properties of the biochars mainly depend upon the feedstocks and pyrolysis conditions such as pyrolysis temperature, speed of temperature increase, pyrolysis pressure and so on.^38^ The physiochemical properties of the three types of feedstock pyrolyzed with different highest treatment temperature are listed in Table 2. Compared with CS and CCS, the SS-derived biochar dramatically increased the ash content (by 42.2–67.3%) and, notably, the ash content of the biochar increased with increasing pyrolysis temperature and there was significantly positive correlation between them. This may indicate that sewage sludge contains higher amounts of inorganic matter, which were concentrated and retained in the biochar during the pyrolysis process.^39^ Qambrani^40^ also reported that sewage sludge biochar has a relatively high ash content. The BET surface area is an important parameter to evaluate the adsorption ability of biochar. The specific surface areas of biochar samples produced from CS (29.8–56.6 m^2^ g^−1^) were significantly higher than those produced from CCS (16.1–26.3 m^2^ g^−1^) and SS (2.32–12.7 m^2^ g^−1^). The lower values for SS biochar than the other two types of biochar could be explained by the fact that SS biochar has a rather high ash content, which would cause the flow channels to downsize and some of the biochar micropores even being completely blocked. Song and Guo,^41^ who examined the BET surface area of poultry-derived biochar, also reported that low specific surface area was due to the biochar micropores being filled or blocked by ash. With increasing pyrolytic temperature, the carbon content of biochar generally increased due to the organic fraction being transformed into carbonized structures during the pyrolysis process, while N, H and O contents decreased. An increase in carbon content and a reduction in H/C value indicated the incorporation of aromatic-containing structures.^42^

**Table 2:** Physicochemical characteristic analysis of different types of biochar produced

Parameter (unit)	CS400	CS500	CS600	CCS400	CCS500	CCS600	SS400	SS500	SS600
Biochar yield (wt%)	41.9	33.2	28.7	38.6	35.2	29.8	50.2	41.8	37.9
Ash content (wt%)	17.1	19.0	20.8	2.5	2.7	3.6	42.2	56.3	67.3
pH	8.2	8.3	8.3	9.3	9.5	9.7	8.7	9.5	11.1
BET surface area (m^2^ g^−1^)	29.8	32.8	56.6	16.1	18.9	26.3	2.32	1.92	12.7
C (wt%)	55.02	64.82	65.31	58.12	66.09	69.88	15.02	16.86	17.63
N (wt%)	0.62	0.38	0.29	1.52	1.29	1.03	2.73	2.27	2.09
H (wt%)	5.12	4.37	2.09	3.92	2.17	1.92	1.83	1.62	1.21
O (wt%)	25.72	19.13	12.82	29.32	27.08	19.82	27.20	26.63	19.08
H/C	0.093	0.067	0.032	0.067	0.033	0.027	0.12	0.096	0.069
O/C	0.47	0.30	0.20	0.50	0.41	0.28	1.81	1.58	1.08

### 3.2. Effect of pyrolysis temperature on characteristics of biochar

The yield of the different types of biochar showed a significant decreasing trend along with an increase in pyrolysis temperature (Table 2). For instance, the yield of CS at 400 °C was 41.9%, while at 600 °C the yield decreased to 28.7%. At temperatures from 400 °C to 600 °C, the yields of CS, CCS and SS were reduced by 31.50%, 22.80% and 24.50%, respectively. Atomic H/C and O/C ratios are used to evaluate the degree of aromaticity and carbonation of different types of biochar.^43^ From the atomic ratios in Table 2, the H/C and O/C ratios of biochar samples decreased with the pyrolysis temperature, indicating a higher degree of aromaticity and associated with high contents of oxygen-containing groups after pyrolysis.^42^ The BET surface area was determined, and the results indicated that specific surface area increased with the increase of pyrolysis temperature. For instance, the CS400 specific surface area increased significantly, from 29.8 to 56.6 m^2^ g^−1^, as the pyrolysis temperature increased from 400 °C to 600 °C, whereas SS600 had a 5.47-fold larger surface area at 600 °C compared to that at 400 °C. Increasing the pyrolysis temperature causing an increase in the surface area could be attributed to the removal of volatile materials after pyrolysis, resulting in the pore volumes of biochar being increased.^43^

### 3.3. Effect of biochar on methane production

#### 3.3.1. Effect of biochar types on methane production

**Fig. 1:**
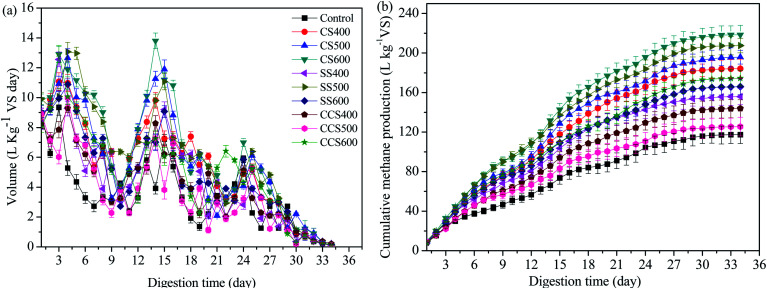
Effect of different types of biochar on (a) the daily methane production and (b) cumulative methane production.

The cumulative and daily methane production yields are presented in Fig. 1. As shown in Fig. 1a, there were three peaks in the daily methane production yield of most groups, though the occurrence time and persistence period of the peaks differed. When the third methane production peak was over, the gas production in all 10 groups gradually stopped, indicating the end of sludge AD. The first methane production peak of all digesters occurred in the first 3–5 days of digestion, which could correspond to the dissolved and easily degradable substances digested by methanogens. And organic acids such as VFAs are gradually generated from the hydrolytic acidification of soluble organic matter. However, the methanogenic activity of methanogens was suppressed when the accumulated amounts of VFAs exceed the regulating ability of biochar, resulting in the methane production rate decreasing significantly. Zhao^44^ also reported AD process inhibition caused by the higher intermediate product concentrations. The occurrence of the second peak of the CS and CCS treatment groups appeared earlier than that of the control and SS treatment groups. The reasons may be that biochar has alkaline groups on its surface, which can neutralize large amounts of organic acids generated in the early stage of AD and alleviate the acid inhibition phenomenon in the system. Secondly, the specific surface areas of CS and CCS are generally larger than that of SS, and such larger specific surface area is suitable for the metabolism and growth activities of methanogens and other microorganism. Finally, CS and CCS may contain nutrients that methanogens can use to promote their activity and increase the conversion efficiency of VFA. Daily methane yield gradually declined along with the decrease of degradable organic matter after the second peak. Afterwards, the methane production rate exhibited a slight recovery from day 24 to 25 for the ten groups, indicating that the organic substances that are difficult to be degraded in the AD system are utilized and decomposed by methanogens to release methane. After that, with the decrease of substances that can be utilized by methanogens, the methane production gradually stopped, and the AD process ends. Interestingly, it was found that sometimes the daily methane yield in the blank groups was higher than in groups with biochar treatment. This phenomenon was due mainly to the accumulated organic acids in digester inhibiting the microbial activity if exceeding the promoted growth rate of methanogens. After a period of adaptation and reproducing of microorganisms, the methanogens gradually adapt to the environment and promote methane production again.^32^

The cumulative methane production yields of the control and test groups are shown in Fig. 1b. The results illustrated that different biomass source and pyrolytic temperature for biochar formation had promoting effects on methane production to different extents. Among all digesters, the maximum methane yield was (218.45 ± 9.55) L per kg VS for CS600, followed by SS500, CS500, CS400, CCS600, SS600, SS400, CCS400, CCS500 and the control, with cumulative methane yields of (207.49 ± 7.29), (195.77 ± 6.92), (184.12 ± 9.69), (174.44 ± 7.72), (165.85 ± 8.02), (155.86 ± 8.19), (143.85 ± 8.92), (125.50 ± 9.36) and (117.36 ± 8.96) L per kg VS, respectively. Compared with the control, the maximum methane yield increased by 86.14% for CS600. Obviously, different types of feedstock and pyrolysis temperature can influence the properties of biochar. Previous studies also reported that aromatization, porosity as well as production method of biochar could influence the effect of biochar on AD.^45^ The average cumulative methane production yield of CS-added digesters was 199.45 L per kg VS, which was 13.07% and 34.83% higher than those of SS-added and CSS-added groups, respectively. Du^46^ found that biochar supplementation during sewage sludge composting could promote the activities of cellulase and peroxidase as well as increasing the microbial diversity. CS had a larger specific surface area than SS and CCS which might be suitable for microorganism growth, and then promoting the release of more cellulase to decompose organic matter during the AD process.^47^

#### 3.3.2. Effect of biochar dose on methane production

**Fig. 2:**
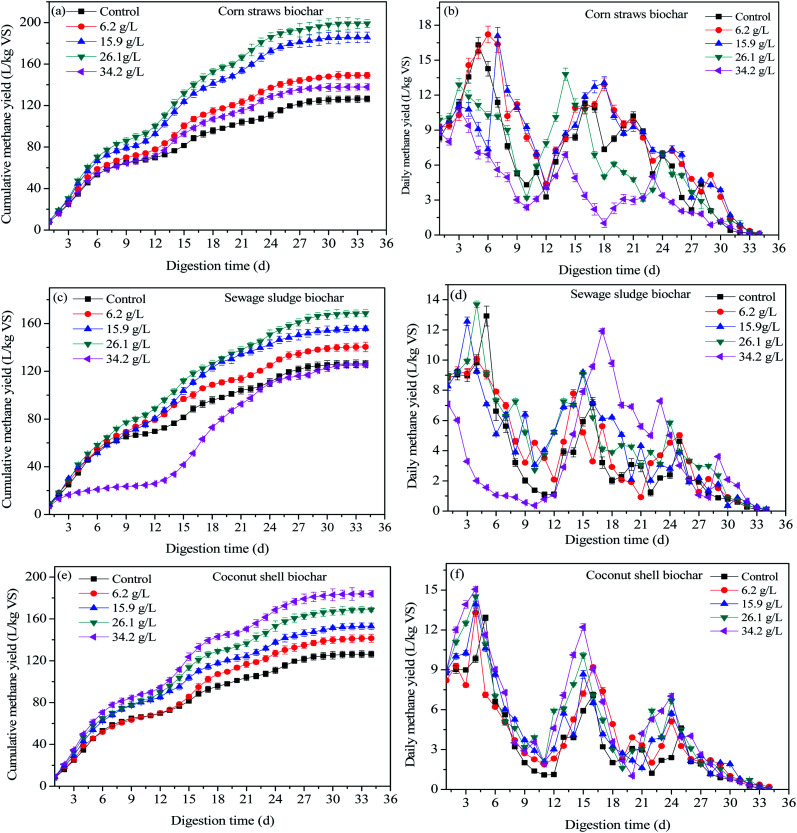
Cumulative methane production (a, c and e) and daily methane production (b, d and f) under different biochar dose conditions.

Experiments showed that different types of biochar had significant effects on accelerating methane production. Besides biochar type, biochar dose can also influence the methane yield.^31^ So, the effects of biochar dose on cumulative methane yield were investigated and the results shown in Fig. 2. Compared with the blank group, the cumulative methane yield increased by 17.80%, 46.99% and 57.47% when the amount of CS biochar added was 6.2, 15.9 and 26.1 g L^−1^, respectively. However, a further increase in the amount of biochar caused a significant decline in the cumulative methane yield, which was 44.26% lower than that for the digesters with 26.1 g L^−1^ treatment (Fig. 2a). A similar situation also occurred with SS biochar (Fig. 2c). The results indicated that at higher supplementation, biochar exhibited obvious inhibitory effects, resulting in lower methane yield. A similar study was conducted by Torri,^48^ who added corn-derived biochar to an AD system and the results suggested that biochar addition not only increased the cumulative methane yield considerably but also improved the reaction rate. Similarly, they also found that the optimal amount of biochar was 10.0 g L^−1^ and greater amounts lowered the methane yield. This was mainly due to the fact that moderate biochar addition could effectively alleviate VFA accumulation resulting in higher levels of methanogenic activity, while higher biochar concentration would lead to more propionic acid accumulated in the digester thereby reducing the AD process stability.^24,49^ A study conducted by Lü^50^ also showed 23.5–47.1% higher methane production in biochar-added digesters in comparison to the control without biochar addition.

For CCS biochar, the cumulative methane yield increased with an increase in additive dose (Fig. 2e). The cumulative methane yields increased by 12.07%, 21.19%, 33.65% and 45.66% in the CCS biochar treatment groups compared with the control group. Moreover, with or without biochar treatment, three rising peaks of daily methane yield were all investigated and adding biochar could cause the methane daily yields to peak much earlier than that of the control group. For instance, CCS biochar showed the three peaks of daily methane yields on the 4th, 15th and 24th day of fermentation, whereas the peaks appeared on the 5th, 16th and 25th day for the control group (Fig. 2f). Biochar exhibits excellent biostability and could also provide nutrition to methanogens during the digestion process, leading to microbial activity enhancement and methane yield increase.^21^

### 3.4. Effect of biochar on pH and VFAs

**Fig. 3:**
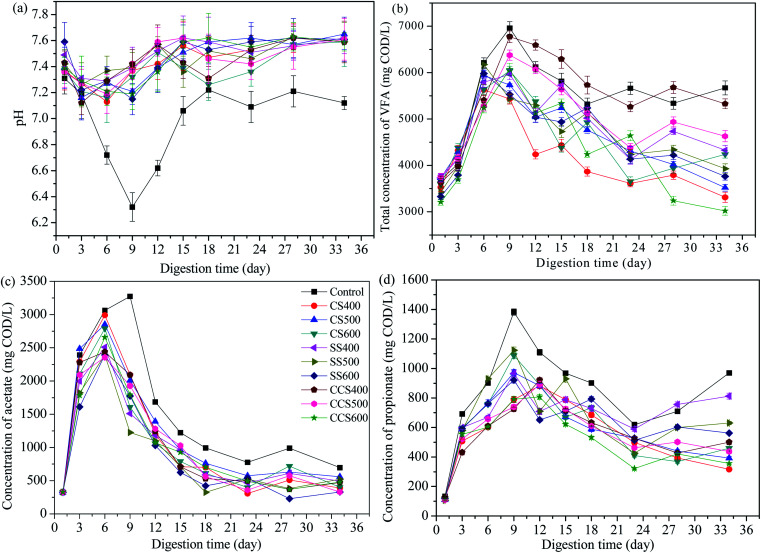
Changes in the pH (a), total VFA (b), acetate (c) and propionate (d) of digestate during digestion time with addition of different types of biochar.

pH is an important parameter to monitor the AD process, and also influences the microbial activity and metabolic pathways. As expected, pH values in digesters with biochar supplementation increased due to the alkaline nature of the biochar (Fig. 3a). Apparently, the pH in all of the groups decreased during the first 6 days of AD, likely caused by the accumulation of VFAs due to the degradation of organics contained in the sewage sludge.^51^ In the control group, the pH dropped from 7.31 to 6.32, while in biochar treatment digesters, the drop in pH was less due to the organic alkali functional groups contained in the biochar.^52^ Subsequently, an upward trend in the pH value was obtained in all digesters due to the consumption of VFAs and the ammonification of protein. For biochar-amended digesters, pH varied in a slightly alkaline range (7.09–7.65), significantly higher than that of the control group (6.32–7.31). Previous studies have reported that the optimum pH range for normal digestion is 6.6–7.6 in an AD system.^53–55^ In the digesters without biochar supplementation, the methanogenic activity was seriously inhibited at a pH lower than 6.6, and then keeping the methane yield at a low level. This means that a more suitable range for microbial activity was obtained due to the buffering capacity of biochar. Thus, biochar plays an important role in the improvement of reactor stability through enhancement of VFA degradation in the digester.

VFAs are the most important intermediate products in AD for producing methane. If the digester accumulates a relatively high content of VFAs, this can lead to the failure of the digestion process. Therefore, the amount of VFAs is considered as an important parameter to evaluate the anaerobic reactor operation status.^56^ The evolution of VFAs during the AD process is shown in Fig. 3b. The total VFA concentration began to climb quickly in all the digesters and reached the highest point during the first 6–9 days of AD. On day 9, VFAs in the control group accumulated to 6962.7 mg COD per L, which resulted in a pH drop (from 7.31 to 6.32) and exerted a slight inhibition of microbial activity. Subsequently, the VFA contents in all digesters had an obvious decreasing tendency because of the removal efficiency of the VFAs being accelerated, indicating that microorganism activity had recovered from various inhibitions. VFA concentration for CS400, CS500, CS600, SS400, SS500, SS600, CCS400, CCS500 and CCS600 reduced by 41.14%, 40.76%, 29.85%, 27.39%, 36.37%, 37.07%, 21.32%, 27.4% and 50.23%, respectively, all higher than that of the untreated digester (18.55%). Furthermore, it can be seen that obvious fluctuations appeared in the control group after 18 days of digestion, resulting in a lower methane yield than the other groups during that period. Thus, it was concluded that biochar addition could alleviate VFA accumulation and stimulate VFA degradation, which were crucial for the stability of the AD process and methane production.

In VFAs, acetate is an important intermediate product for methane production. Similar to the variation of total VFAs, a rapid increase was found in acetate concentration of the test reactors, followed by a sharp decline. The acetic acid concentration reached a maximum level on the 6th day of AD in all the treatment groups, and on day 9 in the blank group. It can be seen that acetate was mainly produced in the initial period of AD, which made up nearly half of total VFAs in the initial phase of digestion (Fig. 3c). The control group generated the highest acetic acid concentration (3275.6 mg COD per L) followed by CS400 and CS500. With biochar-supplemented digesters, the acetate concentration reduced in the range of 80.62–86.98%, between 6 and 34 days of digestion, all higher than that for the digester without biochar treatment (78.76%). Accordingly, the pH value of the blank group was significantly higher than that of the biochar treatment groups, which was in agreement with the results of Alves^29^ who also found that biochar can effectively accelerate the degradation of organic acids. This may be due to the fact that the biochar as additive has large specific area, which serves as a medium for syntrophic VFA-utilizing methanogens to metabolism and promotes rapid utilization of acetate to biogas.^57^

As an important rate-limiting step for methanogenesis, syntrophic metabolism of propionate is relatively slow due to the conversion of propionate to acetate and hydrogen being thermodynamically unfavorable (propionate + 3H_2_O = acetate + HCO_3_^−^ + H^+^ + 3H_2_, Δ*G*_0_ = +76.1 kJ mol^−1^). Dang^58^ has reported that methanogenic bacteria would be inhibited when excess propionate accumulates in anaerobic digesters. During the 34 day AD, the propionate concentration in all digesters rapidly peaked in the initial 9–12 days, and then slowed down (Fig. 3d). The average propionate concentration of the blank group was 838.58 mg COD per L, which was higher than that of all biochar-supplemented digesters (ranging from 512.1 to 665.51 mg COD per L). These results showed that the conversion of propionate worked well with the addition of biochar. Zhao^59^ also found that the addition of biochar in mesophilic digesters could effectively improve the electron-donating and electron-accepting capacity of biochar, resulting in biochar-mediated direct interspecies electron transfer (DIET) promoting the faster metabolism of propionate. Therefore, it could be assumed that biochar supplementation in AD would result in a higher rate of propionate consumption, resulting a higher methanogenic activity and methane yield.

### 3.5. Effect of biochar on ammonia inhibition

**Fig. 4:**
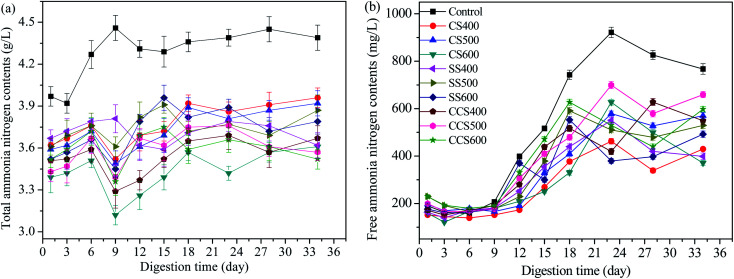
Course of total ammonia nitrogen (a) and free ammonia nitrogen (b) release during the AD process of sewage sludge.

Ammonia inhibition is considered to be one of the prime causes of failure of the AD process.^60^ It has been reported that an ammonia concentration up to 4000 mg L^−1^ could result in the accumulation of VFAs and the inhibition of methanogenic activity, which eventually cause decreased AD stability and methane production.^61,62^ The effects of different biochar types on the variation of total ammonia nitrogen (TAN) concentration were investigated and the results shown in Fig. 4a. Contrasting with the control group, the concentrations of TAN in the biochar-supplemented digesters were significantly lower than that in the digesters without biochar supplementation during the whole digestion period. Typically, the average TAN concentration in biochar treatment digesters was in the range of 3.43–3.76 g L^−1^, which was significantly lower than that of the blank group (4.28 g L^−1^). These results suggested that biochar could effectively alleviate ammonia inhibition and create a suitable environment for methanogen growth. Meanwhile, at higher TAN levels, the cumulative methane yield declined significantly, which suggests that an appropriate TAN concentration range is beneficial to enhance the activity of acidogenic bacteria and improve the organic biodegradation efficiency.^63^ Sharma and Melkania^49^ also reported that a high concentration of TAN caused a clear inhibition of specific enzyme reaction which disturbs AD performance severely. Biochar-amended digesters exhibited a decrease in TAN concentration compared to the control, which was attributed to the fact that biochar has the ability to remove ammonium ions from matrices through adsorption. Similar results were found by Sun,^64^ who reported that biochar addition could enhance AD process stability by alleviating ammonia inhibition and increasing the abundance of the special anaerobic microorganisms. Kizito^65^ also reported 60% ammonium removal from piggery manure with biochar supplementation. The effect of alleviating ammonia inhibition with biochar supplementation depends on digestion environment, particle size of biochar, reactor temperature and some other factors. Further research is needed to explore the optimization of these conditions and resulting better ammonia removal efficiency.

Free ammonia (NH_3_) and ammonium ion (NH_4_^+^) are the two main forms of inorganic ammonia nitrogen in the digesters. pH value is considered as an important parameter used to assess the composition of TAN since it reflects the balance between free ammonia and ammonium ion.^66^ FAN has a greater inhibitory effect than ammonium ion (NH_4_^+^) due to its being highly permeable to the bacterial cell membrane and easily causes pH changes by adsorbing protons in the AD process.^48^ The effect of biochar addition on FAN concentration is shown in Fig. 4b. As shown in Fig. 4b, the FAN concentration in the biochar-amended digesters was obviously lower than in the control group. This indicated that biochar could accelerate the conversion rate of FNA in the AD system which was beneficial to the growth of anaerobic microbes. From day 12 to the end of AD, the digester without biochar supplementation showed sharp fluctuation in its FAN concentrations, showing that biochar addition could effectively increase buffer capacity due to alkaline biochar attenuating pH changes to some extent. Before 23 days, the FAN concentration increased rapidly with a maximum change of 731.87, 310.6, 382.23, 308.77, 388.1, 361.9, 376.33, 345.97, 500.65 and 350.75 in the control and CS400, CS500, CS600, SS400, SS500 SS600, CCS400, CCS500 and CCS600 treatment groups, respectively. Then, the FAN concentration reached a relatively stable value in biochar-supplemented digesters. Under the same condition, the average FAN concentration was 488.93 mg L^−1^ in the blank group, which was 28.53% higher than in the digesters with biochar supplementation. Therefore, comparative analysis of these values indicates that biochar supplementation could effectively maintain the pH in an optimal range for AD, which subsequently will enhance the stability of the digestive process.

### 3.6. Microbial community analysis

**Fig. 5:**
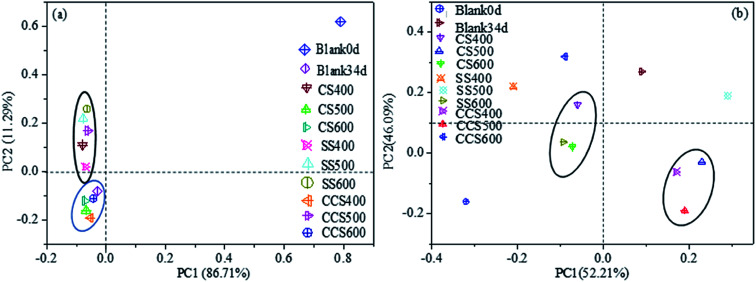
Principal component analysis of bacterial (a) and archaeal (b) communities.

High-throughput sequencing was conducted to evaluate the response of bacteria to biochar supplementation. There were 337 095 high-quality sequence reads obtained across all digesters. A total of 15 926 OTUs were obtained from all the digesters, with a coverage >99% indicating almost complete coverage of diversity. The bacterial and archaeal alpha diversity indexes are exhibited in Table 3. The higher Shannon index and lower Simpson index in biochar-supplemented digesters than those of the blank34d suggested that biochar addition increased the diversity of the microbial community. Principal component analysis (PCA) was also performed to determine the relative differences in the bacterial and archaeal composition. For the bacterial community (Fig. 5a), PCA1 (86.71%) and PCA2 (11.29%) described a total of 98% variation in the bacterial community composition. Meanwhile, component 1 and component 2 accounted for 52.21% and 46.09% for the archaeal community. The PCA result indicated that biochar supplementation had little effect on the bacterial diversity but changed significantly the archaeal community structure.

**Table 3:** Microbial community diversity analysis of bacteria and archaea of different samples

Group	Bacteria			Archaea		
	OTU	Shannon index	Simpson	OTU	Shannon index	Simpson
Blank0d	1325	6.25	0.8925	693	3.92	0.7659
Blank34d	1529	5.72	0.8729	752	3.55	0.7021
CS400	1692	5.89	0.7993	702	3.79	0.6259
CS500	1789	5.92	0.7206	739	3.67	0.6092
CS600	1702	5.99	0.8152	724	3.71	0.6197
SS400	1659	6.09	0.7977	719	3.69	0.6397
SS500	1751	6.16	0.7709	772	3.57	0.6295
SS600	1702	6.19	0.8162	769	3.62	0.6127
CCS400	1609	5.82	0.7917	727	3.75	0.6279
CCS500	1621	5.97	0.7926	759	3.77	0.6392
CCS600	1633	6.12	0.7905	762	3.69	0.6102

Compared with the control, the digesters with biochar treatment showed higher microbial diversity (*p* < 0.05) after 34 days of incubation, which could be explained according to the following three main mechanisms. First, biochar addition can effectively improve the sludge physicochemical properties, such as cation exchange capacity, pH, and moisture content, thus indirectly affecting the microbial activity and diversity during the AD process.^67,68^ Second, biochar can directly provide an available nutrient source to sludge microbes, which could increase their co-metabolisms and proliferation, thereby increasing microbial activity.^69,70^ Third, biochar could be combined with organic amendments in sludge to form organic coating layers to enhance nutrient retention, which should contribute to enhance microbial biomass over time.^71^

**Fig. 6:**
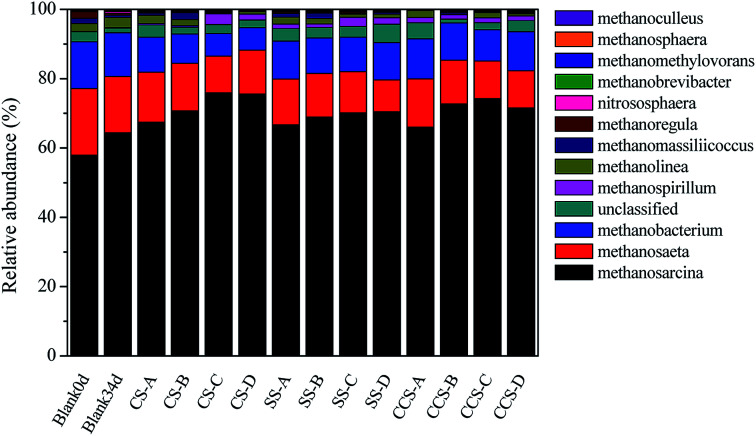
Relative abundance of archaeal community at genus level (A, B, C, and D represent microbial community at different dose: 6.2 g L^−1^, 15.9 g L^−1^, 26.1 g L^−1^ and 34.2 g L^−1^, respectively).

Analyses of archaeal community structures in each reactor with and without biochar were conducted and the results presented in Fig. 6. At genus level, *Methanosarcina* was the most abundant methanogen, followed by *Methanosaeta*, *Methanobacterium* and *Methanospirillum*, respectively. The relative abundance of these four archaeal genera occupied over 92.32% of the total archaeal genera, which were detected in all samples. *Methanosarcina* and *Methanosaeta* are two typical acetoclastic methanogens that can utilize acetate to produce methane.^72^ When CS-, SS- and CCS-derived biochar was supplemented to AD systems, the relative abundance of *Methanosarcina* in digesters had higher proportions of 65.97–75.93%, while the value in the control group increased from 57.92% (day 0) to 64.4% (day 34). In addition to biochar types, changes in biochar dosages could also influence microbial communities. For instance, the relative abundance of genus *Methanosarcina* was significantly increased from 67.45% to 75.93% when the dose of CS-derived biochar increased from 6.2 g L^−1^ to 26.1 g L^−1^. This community shift from *Methanosaeta* to *Methanosarcina* was mainly due to *Methanosarcina* having the ability to utilize multiple nutrients which were boosted in the biochar treatment samples.^73^*Methanosarcina*, which was confirmed as abundant in bound fractions on the surface of biochar, is considered as an important driving factor for acetate degradation which has the ability to translate interspecies electrons directly.^74^ Luo^75^ found that the relative abundance of *Methanosarcina* increased significantly when 10 g L^−1^ biochar was added to the AD system. Recent studies conducted by Eduok^76^ indicated that *Methanosarcina* has remarkable adaption ability to compete with some other specialized methanogens due to its powerful genome. Besides, they also reported that *Methanosarcina* had multiple methanogenic pathways (hydrogenotrophic, aceticlastic and DIET paths) to produce methane compared to other methanogens. This could be another possible reason for the higher cumulative methane yield in biochar treatment digesters in comparison to the control. *Methanobacterium* and *Methanospirillum* are known to be the two methanogens which utilize only hydrogen as a substrate for their growth.^77^ Notably, *Methanobacterium* and *Methanospirillum* had a relatively high abundance in digesters without biochar addition compared with all biochar amendments (12.7% *vs.* 8.78–12.68%). Conclusively, the results demonstrated that biochar supplementation in AD systems could promote the growth of *Methanosarcina* compared with hydrogenotrophic methanogens, which was in accordance with the higher methane production in biochar treatment digesters in comparison to the samples without biochar treatment.

## 4. Conclusions

The effects of the addition of different types of biochar on the AD of sewage sludge were elucidated. Biochar supplementation could facilitate the AD process by creating a surface area for the colonization by microbial cells, accelerating the consumption of organic acids, and alleviating the inhibitory effect of high ammonia nitrogen concentration at mesophilic temperature. Comparing with control, biochar treatment could enhance the cumulative methane yield to different extents under the same conditions. CS600 exhibited a more significant increase in the cumulative methane yield, which was 86.14% higher than that of the control. CS600 was also more effective than the other types of biochar in decreasing TAN concentration during the AD process. Besides, biochar supplementation could selectively enrich the relative abundance of *Methanosarcina* whereas *Methanobacterium* and *Methanospirillum* were inhibited. Overall, biochar as a sustainable alternative material has great potential in digestive engineering due to its cost-effectiveness and excellent functions.

## Conflicts of interest

There are no conflicts to declare.

## Supplementary Material
